# Supplementary material for: The Gut Bacteria Dysbiosis Contributes to Chronic Graft-Versus-Host Disease Associated With a Treg/Th1 Ratio Imbalance
Source: Front Microbiol. 2022 Sep 8;13:813576. doi: 10.3389/fmicb.2022.813576 (PMC9493085; doi:10.3389/fmicb.2022.813576)
Supplement: Supplementary file 1 [file Data_Sheet_1.zip › P101SC18090073-01-B1-3-4_result/04.BetaDiversity/LEfSe/1_MD-vs-MC/biomarkers_raw_images/1_Bacteria-Firmicutes-Clostridia-Clostridiales-Ruminococcaceae.pdf]

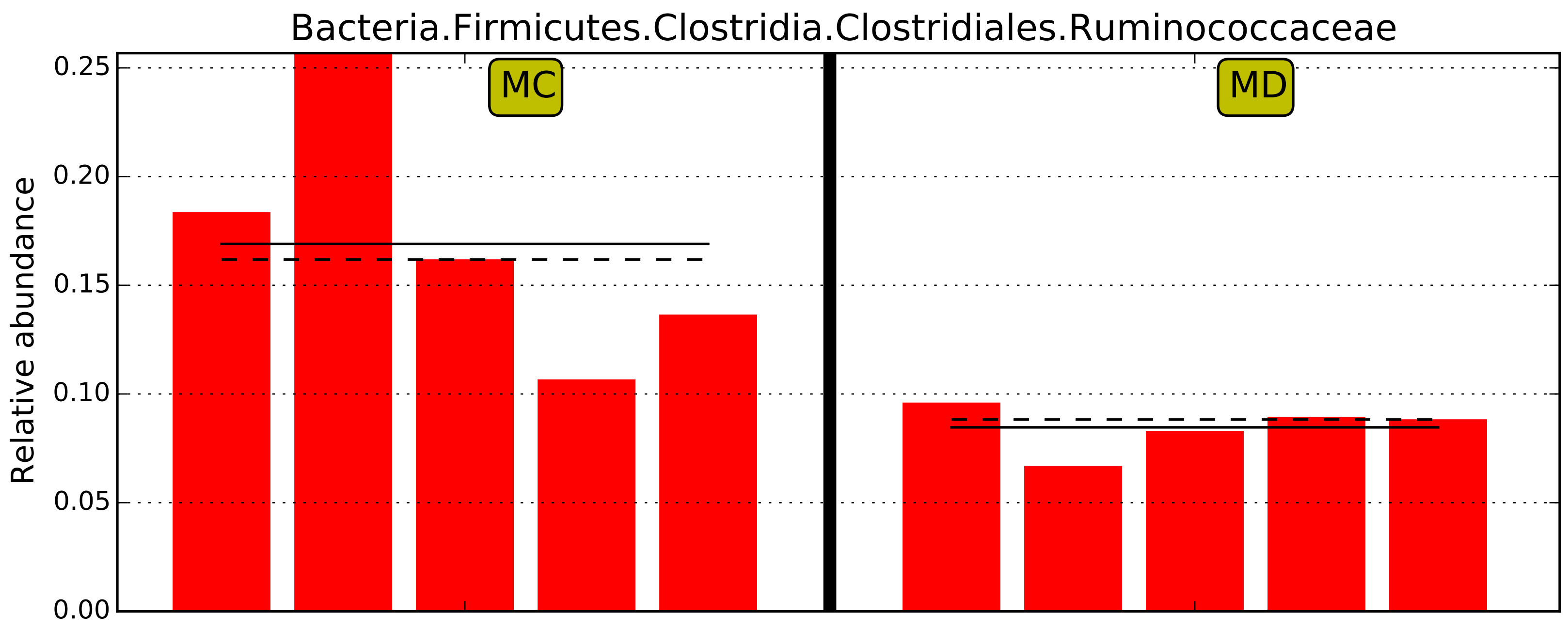

Bacteria.Firmicutes.Clostridia.Clostridiales.Ruminococcaceae
Relative abundance
0.25
0.20
0.15
0.10
0.05
0.00
MC
MD
